# Compliance with the national and WHO antibiotic treatment guidelines for respiratory tract infections and their association with clinical and economic outcomes in Vietnam: an observational study

**DOI:** 10.1093/jacamr/dlaf168

**Published:** 2025-10-09

**Authors:** Vu Quoc Dat, Tran Tat Dat

**Affiliations:** Department of Infectious Diseases, Hanoi Medical University, Hanoi, Vietnam; Hanoi Medical University Hospital, Hanoi Medical University, Hanoi, Vietnam; Department of Infectious Diseases, Hanoi Medical University, Hanoi, Vietnam

## Abstract

**Background and objectives:**

Antibiotic guidelines are a component of antimicrobial stewardship for optimizing antibiotic use. To evaluate the compliance with the national guidelines and the WHO AWaRe Antibiotic Book for the empirical treatment for community-acquired pneumonia (CAP) and acute exacerbations of chronic obstructive pulmonary disease (AECOPD) in critical care units (CCUs) in Vietnam.

**Methods:**

In this 7-day observational study, 51 participating CCUs consecutively enrolled patients aged ≥18 years from March to July 2019. We assessed the compliance for empirical antibiotic prescription using the national guidelines and the WHO AWaRe Antibiotic Book.

**Results:**

We included 500 patients with CAP and 249 patients with AECOPD. The rates of overall compliance with the national guidelines and the WHO AWaRe Antibiotic Book were 54.4% (272/500) and 43.2% (216/500) for CAP; and 48.2% (120/249) and 7.2% (18/249) for AECOPD, respectively. The overall case fatality at 7 days was 4.0% (20/500) in patients with CAP, and 2.0% (5/249) in patients with AECOPD with no significant difference between those receiving compliant and non-compliant regimens by either guideline. The average cost of empirical antibiotic regimens for CAP was lowest at US$3.10 ($3.02-$3.17) per Defined Daily Dose (DDD) for the full compliant regimens versus US$15.26 ($12.72-$17.81) per DDD for the non-compliant regimen according to the WHO AWaRe Antibiotic Book.

**Conclusions:**

Our study indicates that the compliance with the antibiotic guidance was suboptimal in CCUs in Vietnam. Compliance with guidelines for empirical antibiotic therapy could be associated with lower costs.

## Introduction

Non-COVID-19 lower respiratory infection was the major cause of morbidity (4350 episodes per 100 000 population) and mortality (27.7 deaths per 100 000 population) globally, especially in low-income and middle-income countries (LMICs).^1^ In Vietnam, respiratory tract infections were the leading cause of admission to critical care units (CCUs) and accounted for 20.3% of all-cause admission and 12% of total death in CCUs.^2^ The most common cause of community-acquired pneumonia (CAP) were *Streptococcus pneumoniae*, *Haemophilus influenzae* and *Moraxella catarrhalis*^3^ while *Klebsiella pneumoniae*, *H. influenzae*, *Moraxella catarrhalis* and *S. pneumoniae* were the predominant pathogens for the acute exacerbation of chronic obstructive pulmonary disease (AECOPD) with pneumonia.^4^  *S. pneumoniae* isolates from community-acquired infections in Vietnam exhibited high susceptibility to fluoroquinolones (>90%). When using meningitis breakpoints and the Etest, susceptibility rates of *S. pneumoniae* isolates to penicillin and ceftriaxone were only 18.8% and 58.4%, respectively. When using non-meningitis breakpoints, these susceptibility rates to penicillin and ceftriaxone were 97.4% and 92.1% (Etest method), respectively. Among the *H. influenzae* isolates, 67.4% were β-lactamase positive and the susceptibility to fluoroquinolones was <60%.^5^ Among Enterobacteriacae isolates from sputum specimens in patients with CAP, at least 30% of all isolates were resistant to any cephalosporin.^6^

As a life-threating infectious disease, early and adequate empirical antibiotic treatment for CAP is recommended by international guidelines to reduce deaths, often including a β-lactam with or without a macrolide or fluoroquinolone depending on severity of CAP.^7,8^ The degree of compliance with guidelines may be associated with favourable outcomes, such as an improvement in quality of health, a relative risk reduction in mortality of 35% and a shorter hospital stay.^9^ Clinical guidelines should be reviewed every 3–5 years to ensure that they are appropriate and practical, and reflect the most up-to-date available evidence.^10^ However, the development and revision of standard treatment guidelines in lower to middle-income countries is challenging due to the lack of information on local aetiology and antibiotic susceptibility patterns,^11^ and the local adaptation of international guidelines was not performed by standard methodology.^12,13^ In addition, the adaptation of clinical practice guidelines from high-income countries to low- or middle-income country settings can be suboptimal and unrealistic due to the discrepancies in cost effectiveness, resources for implementation and the values and preferences of the targeted audience and patients.

The most recent Vietnamese guidelines for treatment of CAP and AECOPD were released in 2020 and 2018, respectively, heavily referencing the British Thoracic Society, the Infectious Diseases Society of America (IDSA) and the American Thoracic Society (ATS).^14^ However, the earlier recommendations from these international guidelines may have resulted in over-used of cephalosporins, quinolones and macrolides, which triggered MRSA outbreaks.^11^

In December 2022, the WHO AWaRe (Access, Watch, Reserve) antibiotic book was first released to promote the rational empirical antibiotic treatment. Fluoroquinolones were no longer recommended for empirical treatment of CAP in adult patients.^15^ Monitoring of compliance with antibiotic treatment guidelines is one of the activities integrated into antibiotic stewardship interventions^16^ and evaluating the agreement between WHO AWaRe Antibiotic Book and the existing guidelines should be taken into account before deciding on the adaptation of these recommendations as an aspect of applicability. This study aimed to assess the compliance of the empirical antibiotic treatment for respiratory infections according to the national guidelines and the WHO AWaRe Antibiotic Book’s recommendations, as well as its associated clinical outcomes and cost of antibiotic treatment. The secondary aim of this study was to assess the agreement between these two guidelines regarding compliance.

## Materials and methods

### Ethics

The institutional review board at Hanoi Medical University approved the study (59/GCN-DDNCYSH-DHYHN). Eligible patients or their legal representatives provided informed verbal consent.

### Study design and participants

This was a sub-analysis of a 7-day observational study in 51 hospitals in two centrally governed cities (Hanoi and Can Tho) and three provinces (Ha Nam, Thai Nguyen, Kon Tum) in five different ecological regions in Vietnam from March to July 2019 (before the occurrence of COVID-19). We intended to analyse the data collected during this period to exclude the impact of the COVID-19 pandemic on antibiotic prescribing. The study method was described previously.^17^ Briefly, all consecutive patients who had been admitted to the CCUs (Emergency Department, ED and/or ED-based Intensive Care Units) in participating hospitals in these regions were enrolled in this study. In this sub-analysis, we included adult patients admitted to CCUs who had been diagnosed with CAP and AECOPD.

### Data collection

We collected patient’s demographics, diagnoses, clinical data and antibiotic treatment regimens at baseline and the clinical outcome in the 5 days after hospital admission. Cases of CAP and AECOPD were defined by the free-text diagnoses and the International Classification of Disease 10th Edition (ICD-10) code. Pneumonia severity was assessed at baseline using the CURB-65 score (confusion, urea >7 mmol/L, respiratory rate ≥30/min, low systolic (<90 mm Hg) or diastolic (≤60 mm Hg) blood pressure, and age ≥65 years old).^18^ The initial empiric antibiotic regimen was collected within the first 24 hours of admission and the name of antibiotic was recorded according to the Anatomical Therapeutic Chemical (ATC) Classification System.^19^ Antibiotic treatment was assessed according to the Vietnamese guidelines for management of community-acquired infection, AECOPD and the WHO AWaRe Antibiotic Book.^15,20,21^

### Study definition

Empirical antibiotic therapy was classified into full compliance, partial compliance and non-compliance with the national guidelines or the WHO AWaRe Antibiotic Book [see Supplementary material (available as Supplementary data at *JAC-AMR* Online)]. Full compliance was defined as a regimen consisting of the same antibiotic substances (same ATC fifth level code) as recommended, partial compliance, if it consisted of antibiotic substances within the same ATC fourth level and antibiotic class as recommended, and non-compliance, if it consisted of other antibiotics. The full and partial compliance hereinafter were referred to as the overall compliance.

Clinical outcomes at day 7 were defined as deaths (patients who died in the hospital or had a palliative discharge), survival to hospital discharge (patients discharged from hospital) or continued hospital stay (patients who remained in the hospital at day 7). Cost of empirical antibiotic treatment was estimated per DDD per 100 patient-day using the 5-year average price of antibiotic substance (expressed in US dollars/DDD) as previously published.^22^

### Statistical analysis

Data were entered in Epidata (EpiData Association, Odense, Denmark) and analysed using IBM SPSS Statistics for Windows, Version 29.0.0.0 (241) Armonk, NY, USA: IBM Corp. We described categorical variables using frequency distributions and proportions, and described continuous variables using medians and IQRs. The chi square or Mann–Whitney *U*-test was used to compare the differences as appropriate. A Sankey diagram was visualized by SankeyMATIC (https://sankeymatic.com/) and used to show the pattern and the relationship between empirical antibiotic regimens and recommendations by national guidelines and the WHO AWaRe Antibiotic Book. Agreement between the national guidelines or the WHO AWaRe Antibiotic Book in terms of compliance was evaluated using Cohen’s kappa coefficient (*κ*). The strength of agreement of the kappa coefficient was interpreted as follows: ≤0 poor, 0.01–0.2 slight, 0.21–0.4 fair, 0.41–0.6 moderate, 0.61–0.80 substantial and 0.81–1 almost perfect.^23^ Differences were considered statistically significant at two-sided *P* values ≤0.05.

## Results

**Figure 1.**
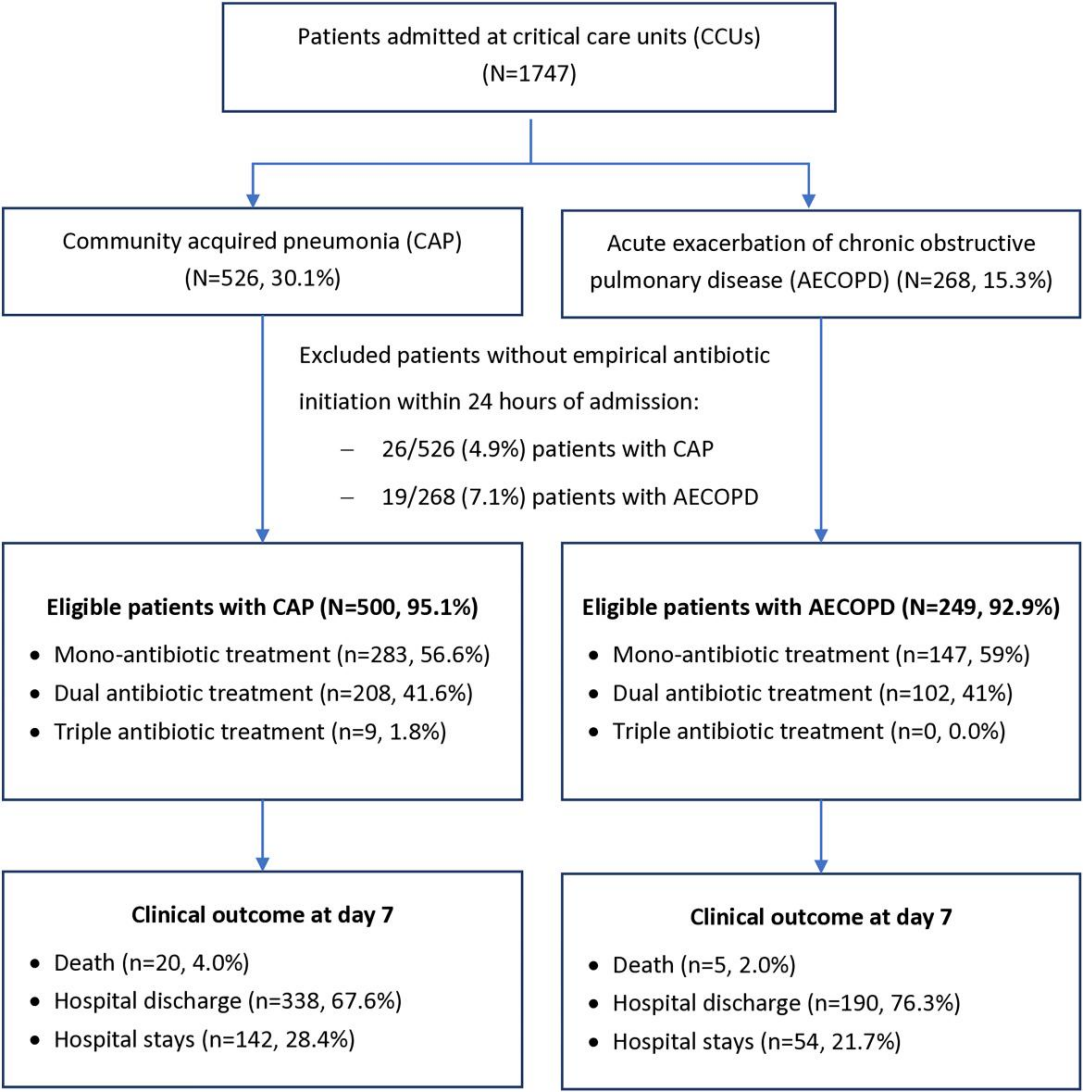
Flowchart of patients included in the study.

During the 7-day observation period, 1747 patients were admitted to CCUs and, among them, there were 526 (30.1%) patients with CAP and 268 (15.3%) patients with AECOPD. Forty-five patients with CAP or AECOPD (5.7%) who did not receive antibiotics within 24 hours of admission were excluded from this analysis (Figure 1). The final analysis included 500 patients with CAP and 249 patients with AECOPD. The proportions of patients with CAP and AECOPD were not significantly different by the level of hospitals (66.6%, 331/479 patients with CAP from primary hospitals versus 67.1%, 169/252 patients with CAP from secondary hospitals, *P* = 0.9). The baseline demographic characteristics, comorbidities and disease severity of included patients are shown in Table 1. In comparison with patients with CAP, patients hospitalized for AECOPD were older, more often male and had higher quick sepsis-related organ failure assessment (qSOFA) scores, but a lower proportion of diabetes and rate of septic shock.

**Table 1.** Demographic characteristics by diagnosis on admission

	All patients (*N* = 749)	Patients with CAP (*n* = 500)	Patients with AECOPD (*n* = 249)	*P* value
Age (median, IQR) (years)	73 (62–83)	72 (59–84)	75 (67–82)	0.04
Level of hospitals				0.899
Primary hospitals, *n* (%)	497 (66.4)	331 (66.2)	166 (66.7)	
Secondary hospitals	252 (33.6)	169 (33.8)	83 (33.3)	
Gender, *n* (%)				<0.001
Female	317 (42.3)	269 (53.8)	48 (19.3)	
Male	432 (57.7)	231 (46.2)	201 (80.7)	
Duration of symptom onset to hospital admission (median, IQR) (days)	2 (1–4)	2 (1–4)	2 (1–5)	0.457
Comorbidity, *n* (%)				
Chronic respiratory diseases	394 (52.6)	156 (31.2)	238 (95.6)	<0.001
Cardiovascular diseases	312 (41.7)	212 (42.4)	100 (40.2)	0.558
Chronic kidney diseases	31 (4.1)	25 (5.0)	6 (2.4)	0.094
Chronic liver diseases	20 (2.7)	10 (2.0)	10 (4.0)	0.107
Malignancy	24 (3.2)	20 (4.0)	4 (1.6)	0.08
Diabetes	76 (10.1)	62 (12.4)	14 (5.6)	0.004
Alcoholism	25 (3.3)	18 (3.6)	7 (2.8)	0.571
Systemic inflammatory response syndrome (SIRS), *n* (%)	572 (76.4)	371 (74.2)	201 (80.7)	0.048
Shock on admission, *n* (%)	34 (4.5)	31 (6.2)	3 (1.2)	0.002
Quick sequential organ failure assessment (qSOFA) score				<0.001
qSOFA 0 and 1, *n* (%)	495 (66.1)	308 (61.6)	187 (75.1)	
qSOFA 2 and above, *n* (%)	254 (33.9)	192 (38.4)	62 (24.9)	
Antibiotic treatment within 24 hours of admission, *n* (%)				0.097
Monotherapy	430 (57.4)	283 (56.6)	147 (59.0)	
Dual therapy	310 (41.4)	208 (41.6)	102 (41.0)	
Triple therapy	9 (1.2)	9 (1.8)	0 (0.0)	
Case fatality at day 7, *n* (%)	25 (3.3)	20 (4.0)	5 (2.0)	0.036

**Figure 2.**
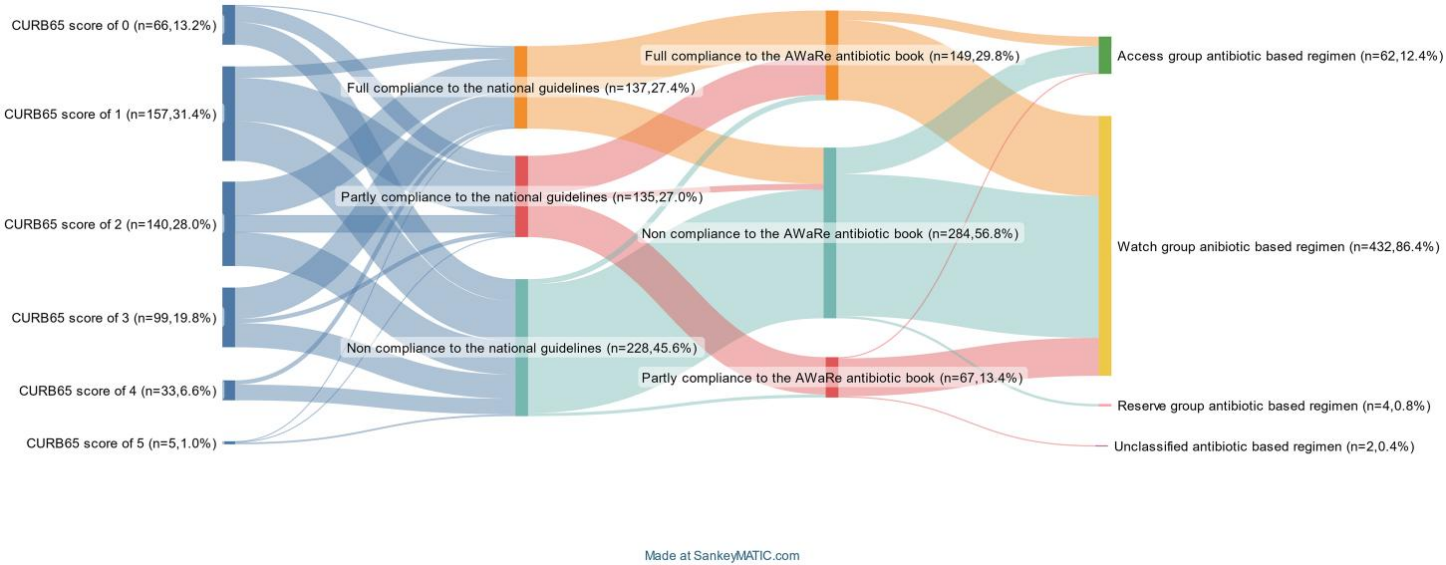
The pattern of antibiotic use and the concordance between Vietnamese national guidelines and the WHO AWaRe Antibiotic Book for treatment of CAP. Vertical bars represent the proportions of patients with CURB-65 scores (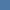), full compliance (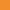), partial compliance (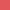) and non-compliance (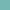). The colour-corresponding curves visually represent the allocation of antibiotic regimen prescriptions across different levels of compliance, as classified according to the national treatment guidelines and the WHO AWaRe Antibiotic Book. The width of each curve is proportional to the quantities of prescriptions associated with that flow: wider curves indicate a higher proportion of patients receiving that regimen.

The rates of overall compliance with the recommendations for CAP and AECOPD were similar according to the national guidelines (54.4% or 272/500 versus 48.2% or 120/249, *P* = 0.11) but significantly different from the WHO AWaRe Antibiotic Book (43.2% or 216/500 versus 7.2% or 18/249, *P* < 0.01). In terms of empirical regimens for CAP, 40.4% (202/500) of regimens were classified as compliant by both the national guidelines and WHO AWaRe Antibiotic Book, and 16.8% (84/500) by only one of the guidelines. For empirical antibiotic regimens for AECOPD, only 7.2% (18/249) of regimens were classified as compliant with both, with 41% (102/249) were classified solely by the national guidelines. The Cohen's kappa (*κ*) statistic for interrater reliability of the classification of the compliance with the national guidelines and the WHO AWaRe Antibiotic Book for CAP was 0.528 (95% CI: 0.467–0.589, *P* < 0.01) (indicating ‘moderate’ agreement) and for AECOPD was 0.076 (95% CI: 0.043–0.109, *P* < 0.01) (indicating ‘slight’ agreement). The Sankey diagram illustrates the agreement between the national guidelines and WHO AWaRe Antibiotic Book for CAP (Figure 2).

The national guidelines identified 27.4% (137/500), 27.0% (135/500) and 45.6% (228/500) of empirical treatment regimens for CAP as fully, partially and non-compliant, respectively, while the WHO AWaRe Antibiotic Book identified 29.8% (149/500), 13.4% (67/500) and 56.8% (284/500) of regimens as fully, partially and non-compliant, respectively. Among regimens for CAP with non-compliance with the national guidelines, 72.8% (166/228) used unclassified antibiotics, 19.3%/(44/228) were classified as over-treatment and 9.9% (18/228) as under-treatment. The non-compliant unclassified regimens consisted of 21.7% (36/166) monotherapy, 74.1% (123/166) dual therapy and 4.2% (7/166) triple therapy. The most frequent non-compliant unclassified regimen was the combination of a third-generation cephalosporin and an aminoglycoside (39/166 or 23.5%), followed by a monotherapy of a second generation cephalosporin (28/166 or 16.9%), followed by a combination of a third-generation cephalosporin and a fluoroquinolone (27/166 or 16.3%).

The proportion of Watch group antibiotic use for CAP between overall compliant and non-compliant regimens was not statistically significant different according to the national guidelines (86.4%, 235/272 versus 88.2%, 201/228, *P* = 0.56) but was statistically significant different according to the WHO AWaRe Antibiotic Book (90.7%, 196/216 versus 84.5%, 240/284, *P* = 0.04). However, the proportions of Access group antibiotic use for CAP were significantly lower in patients who received compliant treatment than in those who received non-compliant treatment according to both the national guidelines (16.2%, 44/272 versus 38.2, 87/228, *P* < 0.01) and the WHO AWaRe Antibiotic Book (8.3%, 18/216 versus 39.8%, 113/284, *P* < 0.01).

The prevalence of monotherapy was higher among patients who were receiving treatment versus not receiving treatment in concordance with the national guidelines (84.2%, 229/272 versus 23.7%, 54/228, *P* < 0.01) and WHO AWaRe Antibiotic Book (99.5%, 215/216 versus 23.9%, 68/284, *P* < 0.01).

**Figure 3.**
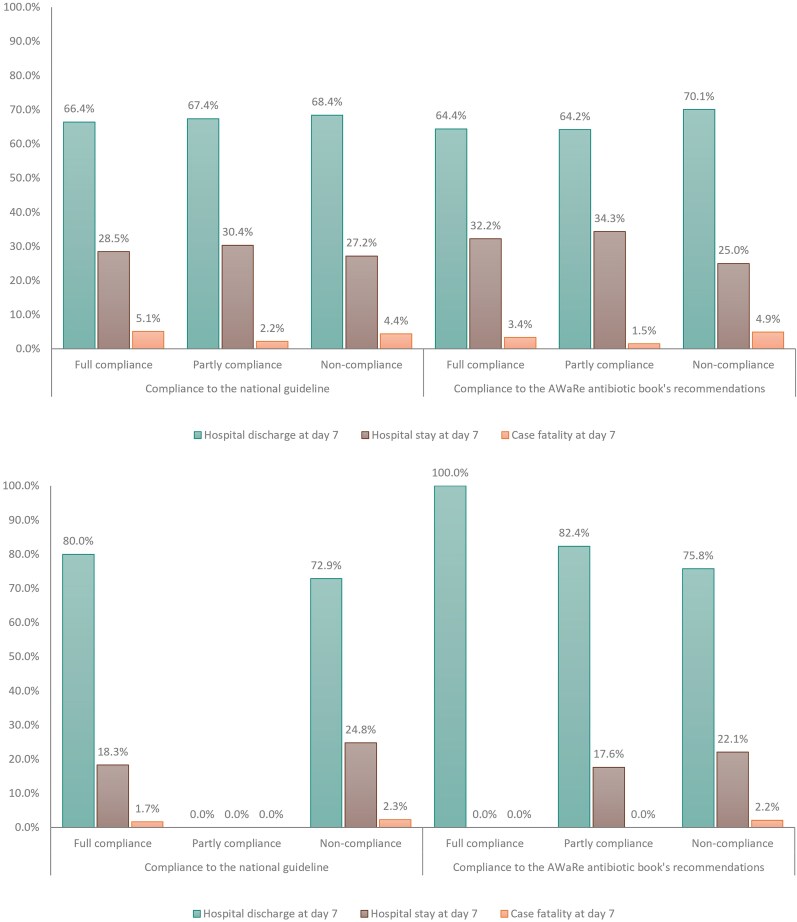
Clinical outcomes according to the degrees of compliance with the recommendations for initially empirical antibiotic use. (a) For CAP. (b) For AECOPD.

The overall case fatality at 7 days was 4.0% (20/500) in patients with CAP and 2.0% (5/249) in patients with AECOPD. The overall case fatality was higher in provincial hospitals than in district hospitals (6.6%, 14/252 versus 2.2%, 11/497, *P* < 0.02) but was not significantly different between those receiving compliant and non-compliant regimens for CAP and AECOPD according to both the national and WHO guidelines (Figure 3).

There were statistically significant differences with regard to average costs of empirical antibiotic regimen per DDD in the three degrees of compliance, with the lowest costs being seen in the full compliant regimens and the highest cost in regimens non-compliant with both the national and WHO guidelines (Table 2).

**Table 2.** Cost of antibiotic regimen (per DDD) by the compliance with the recommendations for initially empirical antibiotic use

Diagnosis	National guideline’s regimen		The WHO AWaRe Antibiotic Book	
	*N* (%)	Mean (95%CI) (US$/DDD)	*N* (%)	Mean (95%CI) (US$/DDD)
1. CAP				
Full compliance	137/500 (27.4)	$6.73 ($5.78–$7.67)	149/500 (29.8)	$3.10 ($3.02–$3.17)
Mono antibiotic therapy	101/137 (73.7)	$5.27 ($4.41–$6.14)	148/149 (99.3)	$3.07 ($3.02–$3.13)
Dual antibiotic therapy	36/137 (26.3)	$10.81 ($8.59–$13.04)	1/149 (0.7)	$6.41
Partial compliance	135/500 (27)	$6.38 ($5.24–$7.51)	67/500 (13.4)	$7.87 ($6.82–$8.93)
Mono antibiotic therapy	128/135 (94.8)	$5.38 ($4.71–$6.06)	67/67 (100)	$7.87 ($6.82–$67.00)
Dual antibiotic therapy	7/135 (5.1)	$24.49 ($9.21–$39.77)	0	−
Non-compliance	228/500 (45.6)	$15.53 ($12.41–$18.66)	284/500 (56.8)	$15.26 ($12.72–$17.81)
Underuse of antibiotics	18/228 (7.9)	$5.43 ($3.54–$7.33)	7/284	$13.79 ($13.79–$13.79)
Overuse of antibiotics	44/228 (19.3)	$18.51 ($14.47–$22.55)	0	—
Unclassified antibiotics	166/228 (72.8)	$15.84 ($11.70–$19.97)	277/284	$15.30 ($12.69–$17.91)
Non-compliance				
Mono antibiotic therapy	54/228 (23.7)	$3.28 ($2.07–$4.48)	68/284	$6.12 ($4.62–$7.62)
Dual antibiotic therapy	165/228 (72.4)	$18.32 ($14.32–$22.32)	207/284	$17.28 ($14.03–$20.54)
Triple antibiotic therapy	9/228 (3.9)	$37.94 ($20.38–$55.49)	9/284	$37.94 ($20.38–$55.49)
2. AECOPD				
Full compliance^a^	120/249 (48.2)	$6.67 ($5.81–$7.54)	1/249 (0.4)	$2.59
Partial compliance^a^	0	−	17/249 (6.8)	$14.25 ($13.28–$15.21)
Non-compliance	129/249 (51.8)	$11.65 ($9.82–$13.48)	231 (92.8)	$8.91 ($7.77–$10.05)
Mono antibiotic therapy	27/129 (20.9)	$2.17 ($0.45–$3.89)	129 (55.8)	$4.76 ($4.03–$5.49)
Dual antibiotic therapy	102/129 (79.1)	$14.16 ($12.15–$16.17)	102 (44.2)	$14.16 ($12.15–$16.17)

^a^All regimens were monotherapy.

## Discussion

This is the first Vietnamese study evaluating compliance with national guidelines and the WHO AWaRe Antibiotic Book for treating respiratory tract infections. Across 51 hospitals, only 54.4% and 48.2% of antibiotic prescriptions for CAP and AECOPD, respectively, were compliant with the national guidelines, even when considering the inclusion of the alternate regimens using the same antibiotic class of recommended drugs. The WHO AWaRe Antibiotic Book shows moderate agreement with the Vietnamese national guidelines for antibiotic treatment of CAP but only slight agreement for AECOPD in classification of compliance. In addition, Watch group antibiotic consumption was high in compliant regimens.

In the global point-prevalence study for 2015 with data from 53 countries, the rate of compliance with local antibiotic guideline was 74.4%.^24^ In a systematic review of 17 studies with 82 240 adult patients with CAP in high and upper middle-income countries, the rate of overall compliance with ATS/IDSA guideline was 65.2% and it was associated with a statistically significant reduction in 30-day mortality rate [crude odds ratio (OR): 0.49, 95%CI: 0.34–0.70] and in-hospital mortality rate (crude OR: 0.63, 95%CI: 0.43–0.92).^25^ However, in three studies with available data of outcomes on ICU admission, the 30-day mortality and in-hospital mortality was not significantly different between patients with and without guideline-concordant therapy.^25^ In a study in China among 262 health facilities between 2017 and 2019, the rate of compliance with the Chinese guidelines for CAP was reported as 69.6%, and elderly patients with CAP were more likely to be prescribed with guideline-recommended antibiotics than children and adults.^26^ We found only one study in Lao PDR that reported the compliance with national guidelines for pneumonia ranged from 56% to 77% in children ≤14 years with only 6% in adults.^27^ Data on the rate of compliance with antibiotic guidelines for CAP and AECOPD are limited in the Southeast Asia region, especially in critical care settings.

With the exception of very severe illness in patients in ICUs, antibiotic prescription for AECOPD has been controversial because AECOPD can be triggered by viral infection, as such, bacterial pathogens are not often identified and the evidence of beneficial effects of antibiotic on mortality and length of stay remains uncertain.^28^ In the WHO AWaRe Antibiotic Book, the antibiotic treatment for AECOPD was limited to oral regimen of amoxicillin, cefaleuxin and doxycyline for mild to moderate cases and amoxicillin + clavulanic acid for severe cases with a caution of risk of infections caused by multidrug-resistant pathogens in patients with frequent exacerbations cases.^15^ Comparing the antibiotic regimen across clinical trials, the AWaRe antibiotic book recommends fewer options for antibiotic treatment and did not include macrolide, piperacillin-sulbactam and ceftazidime.^15,28^ For both CAP and AECOPD, the WHO AWaRe Antibiotic Book did not recommend fluoroquinolones. This was the main difference between the Vietnamese and various international guidelines and may contribute to the low agreement between the national guidelines and the WHO AWaRe Antibiotic Book in the classification of compliance. It is widely accepted that no single empirical antibiotic regimen fits all populations, taking into account local ecological patterns in guidelines is crucial. Currently, the AWaRe antibiotic book contained recommended antibiotics for treating pneumonia but did not cover specific recommendations for bacterial pathogens causing pneumonia in endemic regions, such as Rickettsiales, and *Burkholderia pseudomallei*. Although the true prevalence of Rickettsiales and *B. pseudomallei* causing pneumonia was unknow in Vietnam, it was reported that ricketsial diseases were common among patients with undifferentiated febrile illness (30.8%)^29^ and seroprevalence of meliodosis in Vietnamese residents was 6.4%–31.8%.^30^

In an international, multicentre study of the cost effectiveness of adherence to IDSA/ATS guidelines in 1635 elderly patients with CAP in 12 countries from 2001 to 2007, the compliance with antibiotic guidelines were associated with lower costs when compared with the over-treated group (US$1379 less) and under-treated groups (US$799 less).^31^ In Vietnam, the Social Health Insurance system covers at least 80% of the cost of antibiotics for insured inpatients, with higher coverage—up to 100%—for priority groups such as children under six, individuals living in poverty, ethnic minorities in remote areas and war veterans. Before 1 January 2025, many Watch group antibiotics (e.g. Vancomycin, moxifloxacin) and Reserve group antibiotics (e.g. carbapenems, linezolide…) were only reimbursed at higher-level hospitals (national and provincials) and were not covered at lower-level facilities (district hospitals or community health centres).^32^ A policy change in 2025 introduced a unified drug list, allowing these antibiotics to be reimbursed across all levels of care.^33^

The implementation of the national guidelines may not be effective in Vietnamese hospitals due to the lack of training, clinical auditing, availability of antibiotics and doctor perception of antibiotic effectiveness.^34^ Vietnamese doctors tended to choose the empirical broad-spectrum and combination therapy (carbapenem containing regimen) by clinical severity.^35^ In our previous report, in the same study sites, the proportion of Watch and Reserve group antibiotics for patients admitted to the CCUs were 87.3% and 0.54%, respectively.^17^ The high rate of Reserve and Watch group antibiotic use was aligned with the recommendations of empirical broad-spectrum therapy for adult patients presenting with sepsis or septic shock to cover all likely pathogens in Surviving Sepsis Campaign.^36^ In Vietnam, the availability of Watch and Reserve group antibiotics was highest in national-level hospitals, followed by provincial-level facilities, with district-level hospitals exhibiting the lowest access.^22^ In turn, this availability could influence the selection of empirical regimen. Institution-specific and locally adapted guidelines are necessary as a component of antimicrobial stewardship for increasing the adherence to the algorithm for clinical management of common infections.^10^

Whereas guidelines for empiric antibiotic treatment in LMICs were often inadequate in terms of promoting the use of narrow-spectrum antibiotics and timely updates, the WHO AWaRe Antibiotic Book is an important tool in antimicrobial stewardship. However, given the wide variation in local resistance patterns, countries need to adapt these recommendations to reflect the clinical relevance of the aetiology, local susceptibility patterns, as well as the feasibility, accessibility, and availability of antibiotics.

Our study had several limitations. First, the findings from this study should be generalized to current clinical practice with caution, because our study was conducted before the COVID-19 pandemic and the data of aetiology and antibiotic resistance patterns was unknown in the study sites. The diagnosis of CAP and AECOPD were based on the coded data and chart review, without systemic validation against established diagnostic criteria, which may result in some degree of misclassification. Second, the in-hospital mortality rate among patients in CCUs was influenced by many factors, rather than initially empirical antibiotic treatment. The short follow-up period (7 days) and the inclusion of only initial antibiotic treatment in this study could not accurately assess the specific-cause mortality or all-cause mortality as the outcome of interest. Our analysis focused on patients with AECOPD requiring ICU admission, thus presenting as a subset of patients with critically severe diseases, while the AWaRe antibiotic book recommends options for typical exacerbations. Further controlled studies with better methodological design should be developed to measure the impact of compliance with the guidelines and the AWaRe antibiotic book, as well as their adaptations to clinical outcomes in settings with a higher burden of antibiotic resistance, and in a more generalized context. Third, there was a bias of confounding by indication, wherein patients with more severe diseases were less likely to receive guideline-concordant regimens. This potential bias could lead to an underestimate or overestimate the true association between the guideline compliance and clinical outcome.

In conclusion, we observed a suboptimal compliance with the antibiotic treatment guidelines for respiratory tract infections in Vietnam. There was a potential economic benefit of compliance with the WHO AWaRe Antibiotic Book, but the effectiveness of this guideline implementation should be evaluated in future studies.

## Supplementary Material

dlaf168_Supplementary_Data

## Data Availability

The data that support the findings of this study are available from the author V.Q.D. on reasonable request.

## Supplementary data

Supplementary material available as Supplementary data at *JAC-AMR* Online.
