# Supplementary material for: Compliance with the national and WHO antibiotic treatment guidelines for respiratory tract infections and their association with clinical and economic outcomes in Vietnam: an observational study
Source: JAC Antimicrob Resist. 2025 Oct 9;7(5):dlaf168. doi: 10.1093/jacamr/dlaf168 (PMC12509643; doi:10.1093/jacamr/dlaf168)
Supplement: dlaf168_Supplementary_Data [file dlaf168_supplementary_data.docx]

Supplementary material

Recommended antibiotic regimens for CAP and COPD

# 1. Vietnamese guideline for the diagnosis and management of chronic obstructive pulmonary disease (COPD) (issued with decision no. 2767/QĐ-BYT dated 04/07/2023 by the Minister of Health)

Recommended empirical antibiotic regimen for inpatients with acute exacerbation of COPD (AECOPD)

- No risk of Pseudomonas infection
- Moxifloxacin 400mg once daily, or
- Levofloxacin 500-750mg once daily IV; or
- Ceftriaxone 1-2g x 1-2 times/day or
- Cefotaxime 1-2g x 3 times/day IV
- Risk of P. aeruginosa infection (*) (recent hospitalisation, frequent antibiotic use, P. aeruginosa infection in prior COPD exacerbations or Pseudomonas colonisation)
- Ciprofloxacin 400mg x 2-3 times/day IV;
- Ceftazidime 1-2g x 2-3 times/day IV or cefepime 1-2g x 2-3 times/day IV;
- Piperacillin-tazobactam 4.5g x 3-4 times/day IV;
- Imipenem 500mg x 4 times/day IV or meropenem 1g x 3 IV
- Risk of multi-resistant P. aeruginosa infection

Combine 1 of the drugs for patients at risk of P. aeruginosa infection (*) with:

- Amikacin 15-20mg/kg/day IV, or
- Tobramycin 5-7mg/kg/day IV, or
- Colistin 5mg/kg loading dose, then 2.5mg/kg x 2 times/day IV

# 2. Vietnamese guideline for the diagnosis and management of community acquired pneumonia (CAP) (issued with decision no. 4815/QĐ-BYT dated 20/11/2020 by the Minister of Health)

## 2.1. Moderate CAP – Inpatient (Non-ICU)

- Respiratory fluoroquinolones (Moxifloxacin, Levofloxacin)
- Beta-lactam ± beta-lactamase inhibitor (Cefotaxime, Ceftriaxone, Ampicillin or Amoxicillin + Clavulanic acid or Sulbactam, Ertapenem) combined with a macrolide or fluoroquinolone (IV)

**Note:** For patients at risk of *Pseudomonas aeruginosa* infection, select beta-lactams with anti-Pseudomonal activity.

## 2.2. Severe CAP – Inpatient (ICU)

- Broad-spectrum beta-lactams ± beta-lactamase inhibitors, Ertapenem, Ceftazidime, Ceftriaxone, Cefepime

**Plus one of the following (IV):**

- Respiratory fluoroquinolone
- Macrolide

**Note:**

- If Risk of Pseudomonas aeruginosa Infection: Anti-Pseudomonal beta-lactam (Piperacillin/tazobactam, Ceftazidime, Cefepime, Imipenem, Meropenem, Doripenem) plus Ciprofloxacin/Levofloxacin or aminoglycoside + Azithromycin/Clarithromycin
- If Risk of Community-Acquired MRSA (CA-MRSA): Add one of the following antibiotic: Vancomycin, Teicoplanin or Linezolid

# 3. The WHO AWaRe antibiotic book

## 3.1. Community-acquired pneumonia

### Mild to Moderate Cases

- First Choice: Amoxicillin 1 g q8h ORAL Or Phenoxymethylpenicillin (as potassium) 500 mg (800 000 IU) q6h ORAL
- Second choice: Amoxicillin+clavulanic acid 875 mg+125 mg q8h ORAL Or Doxycycline 100 mg q12h ORAL

### Severe Cases

- First Choice: Cefotaxime 2 g q8h IV/IM Or Ceftriaxone 2 g q24h IV (1 g q24h IM). IF CURB-65 ≥2, CONSIDER ADDING Clarithromycin 500 mg q12h (or ) ORAL IV
- Second Choice: Amoxicillin+clavulanic acid 1 g+200 mg q8h IV (A higher daily dose can be considered: 1 g+200 mg q6h). IF CURB-65≥2, CONSIDER ADDING Clarithromycin 500 mg q12h ORAL (or IV)

## 3.2. Exacerbation of chronic obstructive pulmonary disease

### Mild to Moderate Cases

- First Choice : Amoxicillin 500 mg q8h ORAL
- Second Choice : Cefalexin 500 mg q8h ORAL OR Doxycycline 100 mg q12h ORAL

**Severe Cases :**

- Amoxicillin+clavulanic acid 500 mg+125 mg q8h ORAL
